# Vitamin and Mineral Supplementation and Rate of Gain in Beef Heifers II: Effects on Concentration of Trace Minerals in Maternal Liver and Fetal Liver, Muscle, Allantoic, and Amniotic Fluids at Day 83 of Gestation

**DOI:** 10.3390/ani12151925

**Published:** 2022-07-28

**Authors:** Kacie L. McCarthy, Ana Clara B. Menezes, Cierrah J. Kassetas, Friederike Baumgaertner, James D. Kirsch, Sheri T. Dorsam, Tammi L. Neville, Alison K. Ward, Pawel P. Borowicz, Lawrence P. Reynolds, Kevin K. Sedivec, J. Chris Forcherio, Ronald Scott, Joel S. Caton, Carl R. Dahlen

**Affiliations:** 1 Department of Animal Sciences, University of Nebraska-Lincoln, Lincoln, NE 68588, USA; kacie.mccarthy@unl.edu (K.L.M.); cierrahjk@hotmail.com (C.J.K.); friederike.baumgrtne@ndsu.edu (F.B.); james.kirsch@ndsu.edu (J.D.K.); sheri.dorsam@ndsu.edu (S.T.D.); tammi.neville@ndsu.edu (T.L.N.); alison.ward@ndsu.edu (A.K.W.); pawel.borowicz@ndsu.edu (P.P.B.); larry.reynolds@ndsu.edu (L.P.R.); joel.caton@ndsu.edu (J.S.C.); 2 Center for Nutrition and Pregnancy and Department of Animal Sciences, North Dakota State University, Fargo, ND 58108, USA; 3 Central Grasslands Research and Extension Center, North Dakota State University, Streeter, ND 58483, USA; kevin.sedivec@ndsu.edu; 4 Purina Animal Nutrition LLC, Gray Summit, MO 63039, USA; jforcherio@landolakes.com (J.C.F.); rrscott@landolakes.com (R.S.)

**Keywords:** energy, fetal development, maternal nutrition, protein, trace minerals, vitamins

## Abstract

**Simple Summary:**

Trace minerals play a critical role in several biological functions, being essential for fetal growth and development. Maternal nutrition influences the supply of trace minerals to the fetus, which can lead to long-term consequences on offspring performance and health. Our study shows that a maternal vitamin and mineral supplementation (VTM) pre-breeding and during the first trimester of gestation resulted in greater concentrations of Se, Cu, and Co in the liver of beef heifers and their fetuses. These minerals are an essential component of the antioxidant system, which may have positive impacts on the immune system of the future offspring. In addition, we reported that low rates of gain (LG, 0.28 kg/d) from breeding to d 83 of gestation resulted in greater concentrations of Mo and Co in fetal liver compared with moderate rates of gain (MG, 0.79 kg/d). Having a greater fetal trace mineral reserve may be beneficial for neonates, as the immune system of newborn calves is underdeveloped and minerals play a role in that development. Therefore, our study shows that liver weight and concentrations of hepatic trace minerals are affected by maternal dietary treatments, which may impact the performance and health of the future offspring.

**Abstract:**

We evaluated the effects of vitamin and mineral supplementation (from pre-breeding to day 83 of gestation) and two rates of gain (from breeding to day 83 of gestation) on trace mineral concentrations in maternal and fetal liver, fetal muscle, and allantoic (ALF) and amniotic (AMF) fluids. Crossbred Angus heifers (*n* = 35; BW = 359.5 ± 7.1 kg) were randomly assigned to one of two vitamin and mineral supplementation treatments (VMSUP; supplemented (VTM) vs. unsupplemented (NoVTM)). The VMSUP factor was initiated 71 to 148 d before artificial insemination (AI), allowing time for the mineral status of heifers to be altered in advance of breeding. The VTM supplement (113 g·heifer^−1^·d^−1^) provided macro and trace minerals and vitamins A, D, and E to meet 110% of the requirements specified by the NASEM, and the NoVTM supplement was a pelleted product fed at a 0.45 kg·heifer^−1^·day^−1^ with no added vitamin and mineral supplement. At AI, heifers were assigned to one of two rates of gain treatments (GAIN; low gain (LG) 0.28 kg/d or moderate gain (MG) 0.79 kg/d) within their respective VMSUP groups. On d 83 of gestation fetal liver, fetal muscle, ALF, and AMF were collected. Liver biopsies were performed prior to VMSUP factor initiation, at the time of AI, and at the time of ovariohysterectomy. Samples were analyzed for concentrations of Se, Cu, Zn, Mo, Mn, and Co. A VMSUP × GAIN × day interaction was present for Se and Cu (*p* < 0.01 and *p* = 0.02, respectively), with concentrations for heifers receiving VTM being greater at AI and tissue collection compared with heifers not receiving VTM (*p* < 0.01). A VMSUP × day interaction (*p* = 0.01) was present for Co, with greater (*p* < 0.01) concentrations for VTM than NoVTM at the time of breeding. VTM-MG heifers had greater concentrations of Mn than all other treatments (VMSUP × GAIN, *p* < 0.01). Mo was greater (*p* = 0.04) for MG than LG, while Zn concentrations decreased throughout the experiment (*p* < 0.01). Concentrations of Se (*p* < 0.01), Cu (*p* = 0.01), Mn (*p* = 0.04), and Co (*p* = 0.01) were greater in fetal liver from VTM than NoVTM. Mo (*p* ≤ 0.04) and Co (*p* < 0.01) were affected by GAIN, with greater concentrations in fetal liver from LG than MG. In fetal muscle, Se (*p* = 0.02) and Zn (*p* < 0.01) were greater for VTM than NoVTM. Additionally, Zn in fetal muscle was affected by GAIN (*p* < 0.01), with greater concentrations in LG than MG. The ALF in VTM heifers (*p* < 0.01) had greater Se and Co than NoVTM. In AMF, trace mineral concentrations were not affected (*p* ≥ 0.13) by VMSUP, GAIN, or their interaction. Collectively, these data suggest that maternal nutrition pre-breeding and in the first trimester of gestation affects fetal reserves of some trace minerals, which may have long-lasting impacts on offspring performance and health.

## 1. Introduction

Critical factors affecting fetal growth and development take place in the first trimester of gestation, such as maximal placental growth, differentiation, vascularization, and organogenesis [1]. This period of intense metabolic activity requires an adequate supply of nutrients to the developing fetus. Among the essential nutrients, trace minerals play vital roles in hormone and DNA synthesis, cellular antioxidant defense, membrane electric potential, nerve transmission, metabolism of nutrients, among other biological and physiological processes [2,3]. Maternal nutrient intake is one of the main factors that influence the availability of trace minerals for the fetus, and an inadequate supply of these critical nutrients can have a long-lasting impact on offspring growth and health [3,4]. Therefore, it is important to evaluate the effects of nutritional management strategies on the supply of trace minerals delivered to the fetus.

Despite the importance of maternal nutrition in early pregnancy and throughout the first trimester of gestation, producers tend to neglect supplementation of replacement heifers during this critical period and commonly offer supplementation only during late gestation in order to raise body condition scores before calving [5,6]. Additionally, there is a wide variation in supplementation strategies and whether or not to provide micronutrient supplements [7] and protein/energy supplements [8,9] to replacement beef heifers. Based on these findings, we developed a research model to evaluate the effects of a vitamin and mineral supplementation and two different rates of gain (achieved via protein/energy supplementation) during the first 83 d of gestation on maternal and fetal outcomes. These managerial strategies altered placental expression of nutrient transporters [10] and increased the mass of fetal liver in response to VTM supplementation [11], indicating that substrate availability to the fetus may have been increased. Moreover, we observed an increased concentration of AA in maternal serum and allantoic fluid of beef heifers at d 83 of gestation in response to vitamin and mineral supplementation and rate of gain of 0.79 kg/d [12].

The current experiment aims to complement our previous findings and provide a better comprehension of how maternal vitamin and mineral supplementation (from pre-breeding to day 83 of gestation) and two rates of gain (from breeding to day 83 of gestation) would affect concentrations of trace minerals in maternal and fetal liver, fetal muscle, and allantoic and amniotic fluids. Our hypothesis was that fetal stores of trace minerals would increase in response to maternal vitamin and mineral supplementation and moderate rates of gain.

## 2. Materials and Methods

### 2.1. Ethics Statement

All animal procedures were approved by the North Dakota State University Institutional Animal Care and Use Committee (#A19012).

### 2.2. Animals, Experimental Design, and Dietary Treatments

Crossbred Angus heifers (*n* = 72, initial BW = 359.5 ± 7.1 kg) were initially housed and group-fed at the Central Grasslands Research Extension Center (Streeter, ND, USA). Heifers were stratified by BW and randomly assigned to one of two vitamin and mineral supplementation treatments (VMSUP; supplemented (VTM, *n* = 36) vs. unsupplemented (NoVTM, *n* = 36)), allowing time for the mineral status of heifers to be altered in advance of breeding. Therefore, NoVTM heifers were submitted to a mineral depletion period of at least 71 days, as explained further in the text. Diets were delivered as a total mixed ration (TMR) consisting of triticale hay, corn silage, modified distillers grains plus solubles, ground corn, and if indicated by treatment, VTM premix. The VTM premix was fed at 0.45 kg·heifer^−1^·day^−1^, provided macro and trace minerals and vitamins A, D, and E to meet 110% of the requirements specified by NASEM [2], and consisted of ground corn and a loose VTM supplement (113 g of Purina Wind and Rain Storm All-Season 7.5 Complete, Land O’Lakes, Inc., Arden Hills, MN, USA; and 337 g of a carrier; Table 1 and Table 2). Heifers in the NoVTM treatment received the ground corn carrier at 0.45 kg·heifer^−1^·day^−1^.

Fifty days after initiation of the VMSUP factor, heifers were transported to the North Dakota State University Animal Nutrition and Physiology Center (Fargo, ND, USA), where they were randomly housed in group-pens (23.7 m^2^) with 6 heifers per pen. Thus, a same pen housed VTM and NoVTM heifers. Heifers were individually fed daily in an electronic head gate facility (American Calan; Northwood, NH, USA), where they continued to receive their respective VMSUP treatments until the time of artificial insemination (AI). At this facility, the VTM heifers received a pelleted vitamin and mineral supplement fed at a 0.45 kg·heifer^−1^·day^−1^, consisting of 113 g of the vitamin and mineral supplement and formulated to deliver similar levels of vitamins and minerals that were fed pre-breeding; while the NoVTM heifers received a pelleted carrier product fed at a 0.45 kg·heifer^−1^·day^−1^ with no added vitamins or minerals. The duration of time VTM and NoVTM heifers received their treatments varied according to the AI group they were assigned (i.e., treatments were initiated on the same calendar day, but AI occurred over seven AI group timepoints due to logistical constraints. A detailed description is presented in the companion paper (Menezes et al., 2022 submitted [13]). We achieved our targeted VMSUP delivery of heifers receiving or not receiving VMSUP for at least 60 d before AI (range of 71 to 148 d) to allow time for the mineral status to be altered in advance of breeding so effects would be present on offspring from conception through the gestational target.

All heifers were subjected to a 7-d CO-Synch + CIDR estrus synchronization protocol [14], and AI bred to female sexed semen from a single sire. At the time of AI (d 0 of our experiment), heifers were randomly assigned to one of two rates of gain treatments (GAIN; low gain (LG) 0.28 kg/d or moderate gain (MG) 0.79 kg/d), within their respective VMSUP groups. To achieve the LG, heifers were maintained on the basal TMR and targeted to gain 0.28 kg/d, while heifers on the MG had targeted gains of 0.79 kg/d and were fed the TMR plus a protein/energy supplement (a blend of ground corn, dried distillers grains plus solubles, wheat midds, fish oil, urea, and ethoxyquin; Table 2) at the rate of 0.58% of BW as-fed daily. The supplements were top-dressed over a basal TMR (prairie grass hay, corn silage, and dried distillers grains plus solubles; Table 2). After pregnancy diagnosis and fetal sexing, 35 of the 72 heifers originally enrolled were gestating female fetuses and remained in the experiment. Heifers received treatments until the experiment endpoint of d 83 ± 0.27 after breeding, at which time they were ovariohysterectomized [15].

Thus, the experimental design was a completely randomized design with a 2 × 2 factorial arrangement of treatments, resulting in the following combinations: (1) no vitamin and mineral supplement, low gain (NoVTM-LG; *n* = 9); (2) no vitamin and mineral supplement, moderate gain (NoVTM-MG; *n* = 9); (3) vitamin and mineral supplement, low gain (VTM-LG; *n* = 9); (4) vitamin and mineral supplement, moderate gain (VTM-MG; *n* = 8). Data regarding the actual intake of macro and trace minerals by treatments and a comparison to the NASEM (2016) requirements are presented in Table 3.

### 2.3. Sample Collection and Analysis

#### 2.3.1. Liver Biopsies

Liver biopsies were obtained from heifers at three different time-points: prior to VMSUP factor initiation (71 to 148 days before AI), at the time of AI, and at d 83 (±0.27) of gestation. Heifers were restrained in a squeeze chute, and the liver biopsy site, between the 10th and 11th ribs, was clipped with size 40 blades (Oster; Sunbeam Products Inc., Boca Raton, FL, USA) followed by a subcutaneous injection of lidocaine (3 mL of Lidocaine Injectable-2%; MWI, Boise, ID, USA), scrubbed with betadine, and then scrubbed with 70% ethanol (three times each). A stab incision was made at the targeted site, and a liver sample was collected via a Tru-Cut biopsy trochar (14 g; Merit Medical, South Jordan, UT, USA). The sample was then placed in tubes designed for trace mineral analysis (potassium ethylenediaminetetraacetate; Becton Dickinson Co., Franklin Lakes, NJ, USA) and stored at –20 °C until further analysis.

#### 2.3.2. Fetal Muscle and Liver

Heifers were ovariohysterectomized (d 83 ± 0.27 of gestation) and fetuses harvested and dissected. Samples of fetal liver and muscle were collected adopting the procedures described by Crouse et al. [16]. Samples of the liver and muscle from the left hind limb adjacent to the femur were collected, and approximately 50 mg of the samples were placed in tubes (2 mL), snap frozen on dry ice, and stored at −80 °C until further analysis.

#### 2.3.3. Allantoic and Amniotic Fluids

Allantoic (ALF) and amniotic (AMF) fluids were collected as described by Crouse et al. [17] and Menezes et al. [12]. Briefly, a 22-gauge needle (Medtronic, Minneapolis, MN, USA) was used to penetrate the allantoic and amniotic membranes. The fluids were suctioned with a 10-mL syringe, placed in 2 mL microtubes, snap frozen on dry ice, and stored at −80 °C for further analysis.

#### 2.3.4. Trace Mineral Analysis

For trace mineral analysis, all samples were shipped to the Veterinary Diagnostic Laboratory at Michigan State University, where concentrations of trace minerals were determined via inductively coupled plasma mass spectrometry. The following trace minerals were evaluated in this study: Se, Co, Zn, Mo, Mn, and Co. Abundance of each trace mineral (%) in maternal and fetal tissues and fluids was calculated as a percent of the total concentrations of Se, Cu, Zn, Mo, Mn, and Co in the respective media.

#### 2.3.5. Glutathione Peroxidase Activity

On d 83, maternal blood samples were collected via jugular venipuncture using 10-mL sodium heparin vacutainer tubes (Becton Dickinson HealthCare, Franklin Lakes, NJ, USA) and centrifuged at 1500× *g* and 4 °C for 20 min. After centrifugation, plasma and the buffy coat layers were removed. Two hundred microliters of the erythrocyte-rich fraction was aliquoted into two separate microtubes with 800 mL of ice-cold 18 mΩ water. This mixture was subsequently vortexed for a minimum of 10 s to ensure lysis of red blood cells. Microtubes were then centrifuged at 22,000× *g* and 4 °C for 15 min (Allegra X-22R; Beckman Coulter; Brea, CA, USA). After centrifugation, the supernatant was collected and stored at −80 °C until analysis for GPX activity.

Glutathione peroxidase activity was evaluated in the erythrocyte lysate supernatant using a Glutathione Peroxidase Assay kit (Cayman Chemical Company Inc; Ann Arbor, MI, USA). Frozen samples were thawed and diluted 1:80 in assay buffer. The glutathione peroxidase positive control was included in the kit. Change in absorbance at 340 nm was monitored by a microplate Synergy H1 microplate spectrophotometer (BioTek Instruments, Inc.; Winooski, VT, USA) every minute from 0 to 5 min at 25 °C. The average rate of the reaction was calculated, and the NADPH extinction coefficient of 0.00373 µM^−1^, which was adjusted for pathlength, was used to calculate activity and reported as nmol/min/mL.

### 2.4. Statistical Analysis

Concentrations of trace minerals in maternal liver were analyzed as repeated measures using the MIXED procedure of SAS (SAS Inst. Inc., Cary, NC, USA), with VMSUP, GAIN, day, and their interaction as fixed effects and heifer as the subject. The covariate structures were tested, and the structure with the lowest Akaike information criterion/Bayesian information criterion, Unstructured, was used for all variables analyzed. *p*-values ≤ 0.05 were considered significant, and heifer was considered the experimental unit.

Concentrations of trace minerals in fetal liver, muscle, ALF, and AMF were analyzed using the MIXED procedure of SAS (SAS Inst. Inc.), with VMSUP, GAIN, and their interaction as fixed effects. If no significant interactions were present, then the main effects of VMSUP and GAIN were reported. Means were separated using the LSMEANS procedure of SAS with a Tukey–Kramer adjustment, and *p*-values ≤ 0.05 were considered significant. Heifer was considered the experimental unit for all analyses.

Relationships between trace mineral concentrations in maternal liver, fetal liver, fetal muscle, ALF, and AMF were analyzed using the PROC CORR procedure of SAS to generate Pearson’s correlation coefficients.

## 3. Results

### 3.1. Maternal Liver

Concentrations of Se were affected by a VMSUP × GAIN × day interaction (*p* < 0.01; Table 4). Concentrations of Se were similar for all treatments (*p* = 0.84) prior to initiation of the VMSUP factor, and concentrations remained similar (*p* ≥ 0.68) for the NoVTM treatments (NoVTM-LG and NoVTM-MG) throughout the experiment. For heifers receiving VTM (VTM-LG and VTM-MG), concentrations increased (*p* < 0.001) from the initial sampling to breeding and were greater (*p* < 0.001) than all NoVTM treatments at the time of breeding and at the time of tissue collection. Though concentrations of Se were similar (*p* = 0.90) for VTM-LG heifers at breeding and tissue collection, in VTM-MG heifers Se was reduced (*p* < 0.01) at the time of tissue collection (2.26 ± 0.11 μg/g dry) compared with breeding (3.12 ± 0.14 μg/g dry) but was still greater (*p* < 0.01) than all NoVTM treatments at the time of tissue collection.

Concentrations of Cu were also affected by a VMSUP × GAIN × day interaction (*p* = 0.02). Though all treatments were similar (*p* ≥ 0.91) prior to VMSUP initiation, concentrations were greater at breeding and tissue collection (*p* < 0.01) for heifers receiving VTM compared with heifers receiving NoVTM. The three-way interaction was driven by concentrations of Cu at the time of breeding for VTM-LG being similar (*p* ≥ 0.08) to concentrations in VTM-LG and VTM-MG at the time of tissue collection.

Concentrations of Co were affected by a VMSUP × day interaction (*p* = 0.01). Concentrations of cobalt prior to VMSUP initiation and at breeding were lower (*p* < 0.001) for NoVTM heifers compared with VTM at breeding and at surgery and NoVTM at surgery; whereas VTM prior to VMSUP initiation were intermediate and equal to all other treatment × time combinations (*p* ≥ 0.06).

Concentrations of Mn were affected by a VMSUP × GAIN (*p* = 0.04) interaction, with VTM-MG (11.56 ± 0.35 μg/g dry) having greater (*p* = 0.02) concentrations than VTM-LG (10.07 ± 0.33 μg/g dry), with NoVTM-LG (10.43 ± 0.33 μg/g dry) and VTM-MG (10.47 ± 0.33 μg/g dry) being intermediate and equal to other treatments (*p* ≥ 0.12). Concentrations of Mo were influenced by GAIN (*p* = 0.04), with greater concentrations for MG (3.75 ± 0.09 μg/g dry) than LG (3.49 ± 0.09 μg/g dry) heifers. Concentrations of Zn decreased throughout the experiment (*p* < 0.01), being greater prior to VMSUP initiation (164.24 ± 6.59 μg/g dry) compared with samples collected at time of breeding (125 ± 3.86 μg/g dry) and at d 83 of gestation (121.52 ± 2.89 μg/g dry).

### 3.2. Fetal Liver

No VMSUP × GAIN interactions (*p* ≥ 0.07) were observed for any of the trace minerals in fetal liver (Table 5). Therefore, only significant main effects of VMSUP or GAIN are presented. Concentrations of Se (*p* < 0.01), Cu (*p* = 0.01), Mn (*p* = 0.04), and Co (*p* = 0.01) were greater in the liver of fetuses from VTM compared with NoVTM heifers. Concentrations of Mo and Co were influenced by GAIN (*p* ≤ 0.04 and *p* < 0.01, respectively), with greater concentrations in the liver of fetuses from LG dams. Concentrations Zn were not impacted (*p* ≥ 0.21) by VMSUP, GAIN, or their interaction.

### 3.3. Fetal Muscle

None of the trace minerals measured in fetal muscle were influenced by a VMSUP × GAIN interaction (*p* ≥ 0.07; Table 5). Concentrations of Se and Zn were impacted by VMSUP (*p* = 0.02 and *p* < 0.01, respectively), with greater concentrations in the muscle of fetuses from VTM than NoVTM heifers. Additionally, concentrations of Zn were affected by GAIN (*p* < 0.01), being greater for LG than MG. Concentrations of Cu, Mo, Mn, and Co were not influenced (*p* ≥ 0.07) by VMSUP, GAIN, or their interaction.

### 3.4. Allantoic and Amniotic Fluids

In ALF, Molybdenum was the only trace mineral influenced by a VMSUP × GAIN interaction (*p* = 0.03; Table 6); however, no difference between treatments was observed when LSMeans separation was evaluated (*p* ≥ 0.14). Concentrations of Mo in ALF averaged 57.37 ± 6.23 for VTM-MG, 50.86 ± 5.87 for NoVTM-LG, 42.42 ± 5.87 for VTM-LG, and 38.31 ± 5.87 ng/mL for NoVTM-MG. Concentrations of Se and Co were affected by VMSUP (*p* ≤ 0.01), with greater concentrations for VTM than NoVTM heifers. None of the trace minerals in ALF were influenced by GAIN (*p* ≥ 0.18). Concentrations of Cu, Zn, and Mn were not influenced (*p* ≥ 0.09) by VMSUP, GAIN, or their interaction. In AMF, concentrations of trace minerals were not impacted (*p* ≥ 0.13) by VMSUP, GAIN, or their interaction (Table 6).

### 3.5. Correlations among Tissues and Fluids

Data were examined for correlations among the concentrations of individual trace minerals in maternal liver, fetal liver, fetal muscle, ALF, and AMF on d 83 of gestation. Relationships among ten combinations of tissues and fluids were evaluated, and the correlation coefficient and significance are shown in Table 7. Concentrations of Se (*r* = 0.60), Cu (*r* = 0.56), Mn (*r* = 0.35), and Co (*r* = 0.39) in maternal liver were positively correlated (*p* < 0.01, *p* < 0.01, *p* = 0.04, and *p* = 0.02, respectively) with their concentrations in fetal liver; while concentrations of Zn (*r* =−0.34) in maternal liver were negatively correlated (*p* = 0.05) with concentrations of Zn in fetal liver. Concentrations of Se (*r* = 0.40; *p* = 0.02) and Mn (*r* = 0.35; *p* = 0.04) in maternal liver were positively correlated with Se and Mn concentrations in fetal muscle. Positive correlations were also observed among concentrations of Se (*r* = 0.34; *p* = 0.05) and Co (*r* = 0.52; *p* < 0.01) in maternal liver and ALF. No correlations were observed between concentrations of trace minerals in maternal liver and AMF.

When evaluating the following correlations: fetal liver vs. fetal muscle; fetal liver vs. ALF; fetal liver vs. AMF, we observed positive correlations for Se (*r* = 0.55, *p* < 0.01; *r* = 0.49, *p* < 0.01; *r* = 0.39, *p* = 0.02, respectively). Additionally, concentrations of Cu (*r* = 0.38; *p* = 0.02) and Co (*r* = 0.54; *p* < 0.01) in fetal liver were positively correlated with concentrations of Cu and Co in ALF.

When evaluating correlations of trace minerals between fetal muscle and ALF, a positive correlation was observed only for concentrations of Se (*r* = 0.34; *p* = 0.04). A positive correlation (*r* = 0.37; *p* = 0.03) was observed for concentrations of Mo in fetal muscle and AMF. No correlations were observed for concentrations of trace minerals in ALF and AMF (*p* ≥ 0.21).

### 3.6. Abundance of Trace Minerals in Maternal and Fetal Tissues and Fluids

Copper, Zn, and Mn were the most abundant trace minerals in maternal liver at d 83 of gestation, fetal liver, and fetal muscle (Table 8), while Co, Zn, and Mo were the three most abundant trace minerals in ALF and AMF.

Concentrations of Se and Cu were greater (*p* < 0.01) in fetal liver compared with maternal liver, which was greater (*p* < 0.01) than fetal muscle. Concentrations of Zn were greater in fetal liver compared with maternal liver and fetal muscle. Concentrations of Mo and Mn were greater in maternal liver compared with fetal liver, which was greater (*p* < 0.01) than fetal muscle; while concentrations of Co were greater (*p* < 0.01) in maternal liver compared with fetal liver and fetal muscle.

Concentrations of Se, Mo, and Co were greater (*p* < 0.01) in ALF than AMF, while concentrations of Cu, Zn, and Mn did not differ among the two fluids (*p* ≥ 0.12).

### 3.7. Glutathione Peroxidase Activity

There were no differences in GPX activity due to main effects or interactions (*p* ≥ 0.11). Concentrations of GPX averaged 3866.79 ± 1425.64, 7058.17 ± 1425.64, 5332.3 ± 1425.64, and 3996.11 ± 1425.64 mmol/L for NoVTM-LG, NoVTM-MG, VTM-LG, and VTM-MG, respectively.

## 4. Discussion

To the best of our knowledge, this is the first study to report the impacts of vitamin/mineral and protein/energy supplementation on concentrations of trace minerals in the liver of beef heifers from pre-breeding time points through d 83 of gestation and the subsequent concentrations in multiple fetal compartments. Most studies focus on the form of mineral supplement provided (i.e., sulfate vs. chelate minerals) and their effects on trace mineral concentrations in maternal tissues and fluids during the last trimester of gestation and early lactation [18,19,20,21]. However, due to the key role of trace minerals on several biological processes such as glucose, protein, and lipid metabolism, antioxidant defense, DNA synthesis, and membrane potential [2,3,4], embryonic and fetal development may be directly affected by these micronutrients [4]. Additionally, the first trimester of gestation is a period of intense metabolic activity, characterized by maximal placental growth, differentiation, vascularization, and organogenesis [1]. Further, a recent literature review by Van Emon et al. [3] reported that oocyte, embryo, and the developing fetus are susceptible to changes in maternal mineral status. Therefore, an adequate supply of trace minerals to the developing fetus prior to breeding and during the first trimester of gestation may affect fetal development as well as fetal mineral reserves. Our model mimics common production scenarios where producers would choose whether to provide micronutrient [7] or protein/energy supplements [8,9] to their herds. Resultant data provide clear evidence that the availability of mineral substrate impacts concentrations of minerals that accumulate in several fetal compartments and leads to additional questions regarding mechanisms of mineral transport across various maternal and fetal compartments.

### 4.1. Impact of Maternal Dietary Treatments on Fetal Trace Mineral Reserves

In the present study, a vitamin and mineral supplement was an effective way to increase concentrations of Se, Cu, and Co in maternal and fetal liver. These three trace minerals play an important role in protecting cells against oxidative damages [22]; for instance, Se is an essential component of glutathione peroxidase, Cu is needed for the functionality of the enzyme Cu-Zn superoxide dismutase, while Co is required for the synthesis of cobalamin, which prevents perturbations of the antioxidative–prooxidative balance [4,22]. The developing fetus is especially sensitive to oxidative damage, which may lead to lipid, protein, and polysaccharide oxidation and DNA damage with disruption of apoptosis processes that are highly needed in an appropriate location and temporal pattern during organogenesis [23]. Thus, a maternal vitamin and mineral supplement in early gestation may be beneficial to increase the supply of Se, Cu, and Co to the fetus. In agreement with our findings, McCarthy et al. [24] offered free-choice of the same vitamin and mineral supplement utilized in this study and reported greater liver concentrations of Se, Cu, and Co in cows with high mineral intake compared to cows with low mineral intake. Further, McCarthy et al. [24] did not observe differences in liver concentrations of Mo, Mn, and Zn between the two groups of cows. Similarly, in this study, maternal liver concentrations of Mo were not affected by a vitamin and mineral supplementation; however, concentrations of this mineral responded to GAIN, being greater for MG than LG heifers. In fetal liver, the opposite was observed, where concentrations of Mo were greater for LG than MG fetuses. Experiments in swine [25], rats [26], mice [27], and humans [28] have demonstrated that Mo has the ability to cross the placental barrier. However, it is not clear to what extent the maternal diet influences concentrations of Mo in fetal tissues. Recently, Chernitskiy et al. [29] evaluated trace mineral concentrations in tail switch hair of dams and newborn calves and concluded that the level of Mo accumulated in the calf’s body in the last three months of embryo-fetal development does not depend on the elemental status of its dam. Taken together, Mo transport to the developing fetus is much more complex than simple substrate availability and warrants further investigation.

An interesting finding is the concentration of Mn in response to the synergy between the vitamin and mineral and protein-energy supplements, where VTM-MG heifers had greater concentrations than all other treatments, indicating that hepatic stores of Mn in heifers receiving a VTM supplement increase in response to greater rates of gain. Further, as expected, we observed a greater concentration of Mn in the liver of fetuses from VTM than NoVTM heifers.

In a companion paper to the current report (Menezes et al., 2022 submitted [13]), we reported that fetuses from VTM heifers had greater fetal liver mass than fetuses from NoVTM, while fetuses from LG had greater fetal liver mass (as a % of BW) than MG. As previously mentioned, fetuses from VTM presented greater concentrations of Se, Cu, and Co in the liver, and fetuses from LG had greater hepatic concentrations of Mo. Taken together, these results may be interpreted to imply that a micronutrient supplementation and low rates of gain of 0.28 kg/d during the first trimester of gestation increase liver size and trace mineral reserves of the future offspring, which, considering the pivotal role of the liver in nutrient metabolism and utilization, may result in alterations in offspring performance and health.

Concentrations of Zn in maternal liver decreased throughout the experiment, regardless of vitamin and mineral supplementation or rate of gain. As previously mentioned, similar results were observed by McCarthy et al. [24] when comparing Zn liver concentrations of cows with high or low trace mineral intake. Hackbart et al. [30] also reported no differences in Zn concentrations in liver of dairy cows supplemented or not with trace minerals. On the other hand, Pogge et al. [31] observed greater Zn concentrations in the liver of steers that received a trace mineral injection compared to control animals; however, the authors highlight that even though liver Zn concentration was statistically different between the two treatments, it was not large enough to be biologically important. Even though Zn concentrations reported in this study were within the adequate range (75 to 300 mg/kg DM; [22]), Zn in the liver is not considered a readily mobilizable source of Zn [22]. Thus, it is possible that for heifers receiving a vitamin and mineral supplementation, Zn is stored in other tissues in more readily available forms. In mammalian tissues, the largest percent of zinc can be found in the skeletal muscle, approximately 60%, followed by bone (30%), liver, and skin (5%), and the remaining 5% in other tissues [32]. Even though we did not evaluate concentrations of Zn in maternal muscle, our results show that fetal muscle was the only maternal-fetal structure evaluated where concentrations of Zn were impacted by our treatments, with greater concentrations being found in fetal muscle from VTM than NoVTM heifers, and from LG than MG heifers. Whether or not concentrations of Zn in fetal muscle will further affect offspring muscle yield and carcass characteristics is beyond the scope of this study, but studies in rodent [33] and poultry models [34,35] found that zinc is a signaling molecule that regulates protein kinase phosphorylation, including the stimulation of the mammalian target of rapamycin activity, a molecule that plays a significant role in skeletal muscle development. Gao et al. [35] reported that offspring from hens fed a Zn supplement presented an upregulation of genes associated with skeletal muscle development that are activated by Zn. In addition, those animals presented greater breast muscle yield and larger muscle fiber width than offspring from control hens, which led the authors to speculate that Zn could have been responsible for an increased protein synthesis and decreased protein degradation [35]. Our forthcoming evaluation of transcript abundance in fetal liver and muscle tissues collected from this experiment will provide insight into the molecular implications of our treatments.

### 4.2. Abundance of Trace Minerals in Maternal and Fetal Tissues and Fluids

Selenium, Cu, Mn, and Zn are considered the most limiting trace elements for the normal development of fetuses and neonates [36]. In this study, greater concentrations of Se, Cu, and Zn were observed in fetal liver than in maternal liver at d 83 of gestation, independent of dam nutritional treatment, which may indicate increased requirements of these trace minerals by the developing fetus. These findings, along with a positive correlation between concentrations of these trace minerals in maternal and fetal liver, indicate that the mother favors the transfer of nutrients to the fetus in the first trimester of gestation. Fetal liver stores of trace minerals are very important in the neonate calf, since bovine milk is a poor source of trace minerals, especially Cu [36]. In addition, calves are born with an underdeveloped immune system; therefore, greater hepatic concentrations of the trace minerals Se, Cu, Zn, and Mn may have a positive impact on health status and susceptibility to diseases, as the aforementioned minerals play a pivotal role in the humoral and cell-mediated immune response. Thus, the current line of research should be followed through gestation to evaluate impacts on neonatal and post-natal immune system function.

To the best of our knowledge, this is the first study to report trace mineral composition of ALF and AMF in beef cattle. Our results show that Mo, along with Zn, and Cu were the most abundant trace elements in both fluids. Evidence suggests that in late gestation, the chemical composition of ALF and AMF differ substantially from fetal urine in cattle and sheep [37]. However, before mid-gestation, Wintour et al. [38] and Li et al. [39] reported that all fetal urine is diverted to the ALF compartment via the urachus, suggesting that during this period of gestation, the composition of ALF is heavily influenced by the composition of fetal urine, whereas composition of the AMF most closely resembles that of fetal serum. Considering the role of xanthine oxidase, an Mo-containing enzyme, in converting xanthine to uric acid, and the fact that the main excretion route of Mo is via urine [40], we may assume that at d 83 of gestation, the abundance of Mo in ALF is related to the contribution of fetal urine being diverted to the ALF. Additionally, Menezes et al. [12] observed differences in AA concentrations and abundance in these two fluids, and no correlation between concentrations of individual AA found in ALF and AMF, respectively. Clearly the compositions of AMF and ALF are very different, and mechanisms responsible for these differences may be more complex than the permeability of the amniotic and chorioallantoic membranes to various solutes as proposed by Wintour et al. [37].

### 4.3. GPX Activity

Measuring GPX activity in whole blood or erythrocytes is considered a sensitive indicator of Se status; therefore, this approach was used in the current study. We expected erythrocyte GPX activity would be greater for VTM than NoVTM since the first group received a vitamin and mineral supplement that contained Se; however, our results indicated no difference in GPX activity among the treatment groups. According to a recent publication [41], GPX activity may be best in determining Se deficiency since individuals with adequate Se intake are likely to have maximized GPx activity. In this experiment, neither the VTM nor NoVTM were considered deficient in Se, possibly explaining the lack of difference in GPX activity among treatments.

## 5. Conclusions

In summary, a maternal VTM supplementation resulted in greater concentrations of Se, Cu, Co, and Mn in fetal liver and greater absolute fetal liver weight (as reported in a companion paper to the current report). Mechanisms of transfer of trace minerals to the fetus and subsequent storage in fetal liver appear altered and need to be explored further. Additionally, we observed a greater concentration of Mo and Co in fetal liver, Zn in fetal muscle, as well as greater fetal liver mass (expressed proportionally to BW) in heifers gaining at low rates of gain compared with values from heifers gaining at moderate rates of gain, indicating that mechanisms of enhanced nutrient utilization may be programmed in scenarios of reduced maternal growth rates. Considering that key metabolic activities—such as placentation, organogenesis, and vascularization, take place during the first trimester of gestation and provide the foundation for fetal growth and nutrient exchange throughout gestation—further research is necessary to evaluate the post-natal effects of maternal trace mineral and protein/energy supplementation during gestation, as long-term performance and health may be impacted.

## Figures and Tables

**Table 1:** Micronutrient composition of vitamin and mineral (VTM) supplement ^1^ provided to beef heifers during the first trimester of gestation; company guaranteed analysis.

Item	Assurance Levels	
Minerals ^1^	Min	Max
Calcium, g/kg of DM	135.0	162.0
Phosphorus, g/kg of DM	75.0	-
Sodium Chloride, g/kg of DM	180.0	216.0
Magnesium, g/kg of DM	10.0	-
Potassium, g/kg of DM	10.0	-
Manganese, mg/kg of DM	3600.0	-
Cobalt, mg/kg of DM	12.0	-
Copper, mg/kg of DM	1200.0	-
Iodine, mg/kg of DM	60.0	-
Selenium, mg/kg of DM	27.0	-
Zinc, mg/kg of DM	3600.0	-
Vitamins ^2^, IU/kg of DM		
A	661,500.0	
D	66,150.0	
E	661.5	

^1^ Purina Wind and Rain Storm All Season 7.5 Complete Mineral (Land O’Lakes, Inc., Arden Hills, MN); Ingredients: Dicalcium Phosphate, Monocalcium Phosphate, Processed Grain By-Products, Plant Protein Products, Calcium Carbonate, Molasses Products, Salt, Mineral Oil, Potassium Chloride, Magnesium Oxide, Ferric Oxide, Vitamin E Supplement, Vitamin A Supplement, Lignin Sulfonate, Cobalt Carbonate, Manganese Sulfate, Ethylenediamine Dihydroiodide, Zinc Sulfate, Copper Chloride, Vitamin D3 Supplement, Natural and Artificial Flavors, Sodium Selenite. Post-breeding, the VTM supplement was delivered as a pelleted product fed at 0.45 kg·heifer^−1^·day^−1^ (consisting of 113 g of a mineral and vitamin supplement, formulated to deliver similar levels of vitamins and minerals that were fed pre-breeding and 337 g of a carrier). ^2^ Ingredients: Vitamin A Supplement (proprietary), Vitamin E Supplement (proprietary), Vitamin D3 Supplement (proprietary).

**Table 2:** Nutrient composition of total mixed ration and supplements provided to replacement beef heifers during the first trimester of gestation.

Chemical Composition	Total Mixed Ration ^1^	Supplements		
		NoVTM ^2^	VTM ^3^	Protein/Energy ^4^
Dry Matter, %	53.0	86.6	89.6	87.7
Ash, % DM	11.5	5.3	25.1	2.4
Crude Protein, % DM	9.9	15.6	14.8	17.5
Neutral Detergent Fiber, % DM	65.9	41.9	27.6	19.4
Ether Extract, % DM	1.5	-	-	9.1
Non-Fiber Carbohydrates, % DM	11.1	37.2	32.5	51.6
Mineral Content				
Calcium, g/kg DM	5.74	2.47	50.62	0.30
Phosphorus, g/kg DM	2.05	8.94	22.82	4.59
Sodium, g/kg DM	0.26	0.12	19.44	0.24
Magnesium, g/kg DM	2.83	4.47	5.20	1.96
Potassium, g/kg DM	15.81	14.22	13.15	6.05
Sulfur, g/kg DM	2.25	2.41	4.84	2.57
Manganese, mg/kg DM	121.2	103.9	953.4	26.0
Cobalt, mg/kg DM	0.36	0.14	3.38	0.05
Copper, mg/kg DM	4.8	13.7	285.8	3.6
Selenium, mg/kg DM	0.3	0.4	7.0	0.3
Zinc, mg/kg DM	28.4	130.2	1051.8	35.0

^1^ Proportion of ingredients: prairie grass hay (55%), corn silage (38%), and dried distillers grains plus solubles (7%). ^2^ NoVTM: No vitamin mineral supplement was a pelleted product fed at a 0.45 kg·heifer^−1^·day^−1^ with no added vitamin and mineral supplement. ^3^ VTM: Vitamin mineral supplement was a pelleted product fed at a 0.45 kg·heifer^−1^·day^−1^ (consisting of 113 g of a vitamin and mineral supplement, formulated to deliver similar levels of vitamins and minerals that were fed pre-breeding and 337 g of a carrier). ^4^ Blend of ground corn, dried distillers grains plus solubles, wheat midds, fish oil, urea, and ethoxyquin fed at a rate to achieve a targeted gain of 0.79 kg/d for MG treatment.

**Table 3:** Dietary intake of macro and trace minerals and values recommended by the BCNRM (2016; Nutrient Requirements of Beef Cattle).

Composition	Dietary Treatments				BCNRM (2016) Requirements
	NoVTM-LG ^1^	NoVTM-MG ^2^	VTM-LG ^3^	VTM-MG ^4^	
Macrominerals					
Ca, g/d	25.70	32.98	48.04	50.42	26.30
P, g/d	12.32	23.57	19.07	28.61	14.71
Mg, g/d	13.94	21.03	15.72	20.40	9.80
K, g/d	73.67	103.82	81.42	98.08	48.99
Na, g/d	1.17	1.93	9.09	9.64	5.72
S, g/d	10.63	18.22	12.78	18.47	12.25
Microminerals					
Co, mg/d	1.61	2.12	3.10	3.31	1.22
Cu, mg/d	26.02	38.58	138.34	146.82	81.65
Mn, mg/d	562.73	754.58	967.92	1056.12	326.59
Se, mg/d	1.45	2.38	4.27	4.94	0.82
Zn, mg/d	173.11	273.86	560.82	637.53	244.94

^1^ NoVTM-LG: Total mixed ration, no vitamin and mineral supplement, low gain (0.28 kg/d). ^2^ NoVTM-MG: Total mixed ration, no vitamin and mineral supplement, moderate gain (0.79 kg/d). ^3^ VTM-LG: Total mixed ration, vitamin and mineral supplement, low gain (0.28 kg/d). ^4^ VTM-MG: Total mixed ration, vitamin and mineral supplement, moderate gain (0.79 kg/d). The total mixed ration was made of prairie grass hay (55%), corn silage (38%), and dried distillers grains plus solubles (7%). No vitamin mineral supplement (NoVTM) was a pelleted product fed at a 0.45 kg·heifer^−1^·day^−1^ with no added vitamin and mineral supplement. Vitamin mineral supplement (VTM) was a pelleted product fed at a 0.45 kg·heifer^−1^·day^−1^ (consisting of 113 g of a vitamin and mineral supplement, formulated to deliver similar levels of vitamins and minerals that were fed pre-breeding and 337 g of a carrier). A blend of ground corn, dried distillers grains plus solubles, wheat midds, fish oil, urea, and ethoxyquin was fed at a rate to achieve a targeted gain of 0.79 kg/d for moderate gain treatment (MG).

**Table 4:** Concentrations of trace minerals in maternal liver at three different time-points as influenced by maternal vitamin and mineral supplementation (VMSUP) and two different rates of gain (GAIN; low rate, 0.28 kg/d (LG) or moderate rate, 0.79 kg/d (MG).

Trace Mineral Concentration, μg/g Dry	NoVTM ^1^		VTM ^2^		SEM ^4^	*p*-Values						
	LG	MG ^3^	LG	MG ^3^		VMSUP	GAIN	Day	VMSUP × GAIN	VMSUP × Day	GAIN × Day	VMSUP × GAIN × Day
Selenium												
Prior to VMSUP ^5^	2.05 ^a^	1.47 ^a^	1.94 ^a^	1.72 ^a^	0.25	<0.01	0.02	<0.01	0.79	<0.01	0.05	<0.01
Breeding ^6^	1.76 ^a^	1.56 ^a^	2.98 ^c^	3.12 ^c^								
Surgery ^7^	1.64 ^a^	1.54 ^a^	2.87 ^c^	2.26 ^b^								
Iron												
Prior to VMSUP	208.95	230.32	214.12	217.98	49.3	0.17	0.51	<0.01	0.56	0.28	0.02	0.47
Breeding	375.87	348.74	275.35	317.63								
Surgery	347.97	261.66	298.21	246.19								
Copper												
Prior to VMSUP	83.69 ^a^	50.1 ^a^	112.28 ^a^	66.55 ^a^	29.56	<0.01	0.33	<0.01	0.73	<0.01	0.05	0.02
Breeding	66.82 ^a^	38.88 ^a^	195.28 ^b^	218.04 ^c^								
Surgery	39.35 ^a^	27.35 ^a^	196.27 ^b^	184.21 ^b^								
Zinc												
Prior to VMSUP	139.9	173.54	165.46	178.07	13.76	0.27	0.15	<0.01	0.78	0.62	0.23	0.39
Breeding	120.53	123.27	122.68	133.65								
Surgery	119.49	120.37	121.95	123.93								
Molybdenum												
Prior to VMSUP	3.38	3.63	3.68	4.05	0.2	0.52	0.04	0.06	0.33	0.06	0.19	0.83
Breeding	3.57	3.47	3.33	3.57								
Surgery	3.58	3.85	3.39	3.95								
Manganese												
Prior to VMSUP	9.88	10.51	11.01	11.83	0.66	0.29	0.03	<0.01	0.04	0.18	0.65	0.38
Breeding	11.48	11.04	10.74	11.99								
Surgery	9.94	9.86	8.46	10.85								
Cobalt												
Prior to VMSUP	0.14	0.15	0.17	0.19	0.02	<0.01	0.91	<0.01	0.92	0.01	0.36	0.32
Breeding	0.17	0.16	0.22	0.23								
Surgery	0.2	0.19	0.24	0.21								

^1^ NoVTM: No vitamin and mineral supplement was a pelleted product fed at a 0.45 kg·heifer^−1^·day^−1^ with no added vitamin and mineral supplement. ^2^ VTM: Vitamin mineral supplement was a pelleted product fed at a 0.45 kg·heifer^−1^·day^−1^ (consisting of 113 g of a vitamin and mineral supplement, formulated to deliver similar levels of vitamins and minerals that were fed pre-breeding and 337 g of a carrier). ^3^ Heifers fed a pelleted blend of ground corn, dried distillers grains plus solubles, wheat midds, fish oil, urea, and ethoxyquin fed at a rate to achieve a targeted gain of 0.79 kg/d. ^4^ NoVTM-LG (*n* = 9); NoVTM-MG (*n* = 9); VTM-LG (*n* = 9); VTM-MG (*n* = 8). ^5^ Liver biopsy was performed before VMSUP factor initiation (71 to 148 d before artificial insemination). ^6^ Liver biopsy was performed at the time of artificial insemination. ^7^ Liver biopsy was performed at the time of ovariohysterectomy (d 83 ± 0.27 after artificial insemination). ^abc^ Means lacking common superscript differ (*p* ≤ 0.05).

**Table 5:** Concentrations of trace minerals in fetal liver and fetal muscle at d 83 of gestation as influenced by maternal vitamin and mineral supplementation (VMSUP) and two different rates of gain (GAIN; low rate, 0.28 kg/d (LG) or moderate rate, 0.79 kg/d (MG)).

Mineral Concentration, μg/g Dry	NoVTM ^1^		VTM ^2^		SEM ^4^	*p*-Value		
	LG	MG ^3^	LG	MG ^3^		VMSUP	GAIN	VMSUP × GAIN
Fetal Liver								
Selenium	4.23	4.25	6.25	6.39	0.46	<0.01	0.86	0.89
Copper	246.01	277.84	298.21	348.91	22.75	0.01	0.08	0.68
Zinc	440.61	448.24	541.2	563.76	85.35	0.21	0.85	0.93
Molybdenum	0.37	0.33	0.36	0.33	0.02	0.79	0.04	0.81
Manganese	5.09	4.78	5.19	6.03	0.32	0.04	0.39	0.07
Cobalt	0.07	0.05	0.09	0.06	0.01	0.01	<0.01	0.27
Fetal Muscle								
Selenium	0.62	0.67	0.73	0.73	0.03	0.02	0.55	0.41
Copper	5.75	7.15	6.35	5.94	0.49	0.54	0.32	0.07
Zinc	75.23	75.10	85.04	83.78	61.36	<0.01	<0.01	0.99
Molybdenum	0.13	0.15	0.13	0.11	0.01	0.21	0.86	0.07
Manganese	1.00	1.00	1.00	1.31	0.15	0.28	0.28	0.28
Cobalt	0.06	0.08	0.08	0.06	0.01	0.78	0.77	0.12

^1^ NoVTM: No vitamin and mineral supplement was a pelleted product fed at a 0.45 kg·heifer^−1^·day^−1^ with no added vitamin and mineral supplement. ^2^ VTM: Vitamin mineral supplement was a pelleted product fed at a 0.45 kg·heifer^−1^·day^−1^ (consisting of 113 g of a vitamin and mineral supplement, formulated to deliver similar levels of vitamins and minerals that were fed pre-breeding and 337 g of a carrier). ^3^ Heifers fed a pelleted blend of ground corn, dried distillers grains plus solubles, wheat midds, fish oil, urea, and ethoxyquin fed at a rate to achieve a targeted gain of 0.79 kg/d. ^4^ NoVTM-LG (*n* = 9); NoVTM-MG (*n* = 9); VTM-LG (*n* = 9); VTM-MG (*n* = 8).

**Table 6:** Concentrations of trace minerals in allantoic fluid and amniotic fluid from beef heifers at d 83 of gestation as influenced by maternal vitamin and mineral supplementation (VMSUP) and two different rates of gain (GAIN; low rate, 0.28 kg/d (LG) or moderate rate, 0.79 kg/d (MG)).

Mineral Concentration, ng/mL	NoVTM ^1^		VTM ^2^		SEM ^4^	*p*-Value		
	LG	MG ^3^	LG	MG ^3^		VMSUP	GAIN	VMSUP × GAIN
Allantoic Fluid								
Selenium	12.03	10.39	15.03	18.22	1.29	<0.01	0.53	0.06
Copper	24.74	25.31	30.21	31.94	0.01	0.09	0.74	0.87
Zinc	50.00	50.00	62.33	50.00	5.19	0.22	0.22	0.22
Molybdenum	50.86	38.31	42.42	57.37	6.23	0.38	0.84	0.03
Manganese	1.00	1.00	1.02	1.00	0.01	0.34	0.34	0.34
Cobalt	0.19	0.12	0.30	0.29	0.03	<0.01	0.18	0.30
Amniotic Fluid								
Selenium	1.49	1.48	1.61	1.61	0.09	0.16	0.93	0.93
Copper	20.00	20.00	20.00	32.23	5.81	0.28	0.28	0.28
Zinc	50.49	53.74	50.82	50.00	2.08	0.39	0.55	0.31
Molybdenum	6.72	6.47	6.15	5.43	0.53	0.13	0.35	0.65
Manganese	1.53	1.00	1.64	1.00	0.37	0.88	0.13	0.88
Cobalt	nd	nd	nd	nd	-	-	-	-

^1^ NoVTM: No vitamin and mineral supplement was a pelleted product fed at a 0.45 kg·heifer^−1^·day^−1^ with no added vitamin and mineral supplement. ^2^ VTM: Vitamin mineral supplement was a pelleted product fed at a 0.45 kg·heifer^−1^·day^−1^ (consisting of 113 g of a vitamin and mineral supplement, formulated to deliver similar levels of vitamins and minerals that were fed pre-breeding and 337 g of a carrier). ^3^ Heifers fed a pelleted blend of ground corn, dried distillers grains plus solubles, wheat midds, fish oil, urea, and ethoxyquin fed at a rate to achieve a targeted gain of 0.79 kg/d. ^4^ NoVTM-LG (*n* = 9); NoVTM-MG (*n* = 9); VTM-LG (*n* = 9); VTM-MG (*n* = 8). nd = not detected.

**Table 7:** Correlation coefficients for individual trace minerals between maternal liver (Mliver), fetal liver (Fliver), fetal muscle (Fmuscle), allantoic (ALF), and amniotic (AMF) fluids from beef heifers at d 83 of gestation.

Trace Minerals	Mliver vs. Fliver	Mliver vs. Fmuscle	Mliver vs. ALF	Mliver vs. AMF	Fliver vs. Fmuscle	Fliver vs. ALF	Fliver vs. AMF	Fmuscle vs. ALF	Fmuscle vs. AMF	ALF vs. AMF
Selenium	0.60 *	0.40 *	0.34 *	0.23	0.55 *	0.49 *	0.39 *	0.34 *	0.18	0.22
Copper	0.56 *	−0.003	0.31	0.19	0.31	0.38 *	0.07	0.09	−0.06	0.01
Zinc	−0.34 *	−0.19	0.14	−0.19	0.19	−0.02	0.11	0.2	−0.26	0.01
Molybdenum	−0.12	0.09	−0.01	−0.16	0.12	−0.02	−0.01	0.21	0.37 *	0.15
Manganese	0.35 *	0.35 *	−0.01	0.18	0.18	0.06	−0.22	−0.03	−0.05	−0.04
Cobalt	0.39 *	0.06	0.52 *	nd	0.11	0.54 *	nd	−0.06	nd	nd

* Asterisks denote significant correlation (*p* ≤ 0.05). nd = not detected.

**Table 8:** Abundance of trace minerals in maternal liver, fetal liver, fetal muscle, allantoic fluid and amniotic fluid of beef heifers on d 83 of gestation ^1^.

		Tissues				Fluids	
Trace Minerals	Maternal Liver, μg/g Dry	Fetal Liver, μg/g Dry	Fetal Muscle, μg/g Dry	*p*-Value	Allantoic Fluid, ng/mL	Amniotic Fluid, ng/mL	*p*-Value
Selenium	2.07 ^a^	5.25 ^b^	0.69 ^c^	<0.01	13.79 ^d^	1.55 ^e^	<0.01
Copper	109.73 ^a^	291.14 ^b^	6.31 ^c^	<0.01	27.94 ^d^	22.79 ^d^	0.12
Zinc	121.46 ^a^	496.59 ^b^	79.67 ^a^	<0.01	53.17 ^d^	51.30 ^d^	0.49
Molybdenum	3.68 ^a^	0.35 ^b^	0.13 ^c^	<0.01	46.95 ^d^	6.22 ^e^	<0.01
Manganese	9.75 ^a^	5.25 ^b^	1.07 ^c^	<0.01	1.01 ^d^	1.30 ^d^	0.12
Cobalt	0.21 ^a^	0.07 ^b^	0.07 ^b^	<0.01	0.22 ^d^	0.10 ^e^	<0.01

^1^ Average of trace mineral concentrations. ^a–c^ Within a row, least square means without a common superscript differ (*p* < 0.05) due to type of tissue. ^d–e^ Within a row, least square means without a common superscript differ (*p* < 0.05) due to type of fluid.

## Data Availability

The data presented in this study are available on request from the corresponding author.
